# The illusion of having a large virtual body biases action-specific perception in patients with mild cognitive impairment

**DOI:** 10.1038/s41598-021-03571-7

**Published:** 2021-12-15

**Authors:** Hokyoung Ryu, Kyoungwon Seo

**Affiliations:** 1 Graduate School of Technology and Innovation Management, Hanyang University, Seoul, South Korea; 2 Department of Applied Artificial Intelligence, Seoul National University of Science and Technology, Seoul, South Korea

**Keywords:** Dementia, Diagnostic markers, Translational research

## Abstract

The illusion of having a large body makes us perceive objects as smaller than they really are. This action-specific perception effect occurs because we perceive the property of an object (i.e., size) differently according to our unique action capability (i.e., the affordance of body size). Although the body-ownership illusion contributing to this action-specific perception has been studied, its effects remain unclear in neurological patients. We examined the action-specific perception impairments of MCI patients by means of body-ownership illusion in a non-immersive virtual reality environment. Twenty healthy young adults, 21 healthy older adults, and 15 MCI patients were recruited. We assessed their “original-body action-specific perception” and “enlarged-body action-specific perception” using the original and enlarged sizes of their virtual bodies, respectively. The MCI patients’ original-body action-specific perception was no different than that of the healthy controls (*p* = 0.679). However, the enlarged-body action-specific perception of the MCI patients was significantly biased (*p* < 0.001). The inclusion of the enlarged-body action-specific perception provides additional discriminative power for early diagnosis of MCI (89.3% accuracy, 75.0% sensitivity, 100.0% specificity, and 87.5% balanced accuracy).

## Introduction

Researchers are trying to answer whether our visual perception of objects is objective or simply an illusion^1^. To better understand vision, Goodale and Milner^2^ explored two different visual pathways: vision-for-perception and vision-for-action. Vision-for-perception is what we commonly think of as vision, which constructs our perceptual representations of the visual world and the objects within it. In contrast, vision-for-action relates to the visual perception that occurs when we control actions with our body, such as reaching for or grasping an object (for a detailed discussion of this idea, see^3^. Indeed, our body’s actions have a huge impact on our visual perception of an object. According to Gibson’s ecological approach^4^, humans do not perceive the absolute properties of an object. Instead, humans perceive how the object is related to them and what it can afford them. Gibson defined this as *action-specific perception*, in which we perceive an object’s properties, such as its size and one’s distance to it, differently depending on our unique action capabilities^5,6^. For example, Linkenauger et al.’s experiment^7^ confirmed that humans perceive the same objects to be smaller when their grasping hand was enlarged as compared to when the hand appears to be its original size. This finding shows that visual perception of an object is closely related to one’s action capability (in this case, the affordances of body size) to the object. In other words, our action capability relative to an object plays an important reference role in action-specific perceptions^8^.

The body-ownership illusion is essential to study action-specific perception, such as through the modulation of an individual’s action capability to an object^9^. The body-ownership illusion refers to the illusion of owning a part of a body, or an entire body, that is other than one’s own^10,11^. For instance, the classic rubber hand illusion described well how tactile sensations are transferred to an alien limb^12^. The body-ownership illusion induced by the rubber hand illusion affects not only perceptual tasks, but also perceptual scaling judgments in motor tasks, such as grasping an object^13,14^.

Virtual reality (VR), a computer-generated simulation of an interactive three-dimensional environment, can be used to experimentally induce the body-ownership illusion by modulating an individual’s action capability (e.g., the affordances of a virtual body size) to an object. VR provides a controllable, reproducible, and ecological way to study the mechanisms of action-specific perceptions^15,16^. VR can be presented in a first-person perspective (i.e., the virtual body is co-located with the real body^17^) or a third-person perspective (i.e., the virtual body is separated from the real body^18,19^). From a first-person perspective, the body-ownership illusion can be induced by physically touching the participant’s real body while providing visual feedback of touching the virtual body (i.e., visuo-tactile synchrony). From a third-person perspective, the illusion can be induced by the participant seeing their virtual body, projected on a large screen, move simultaneously with their real body (i.e., visuo-motor synchrony^11,17^). Lugrin et al.^20^ named this third-person perspective a non-immersive VR fake mirror in which the participant’s body size, position, and movement were faithfully reproduced by a virtual body. The reflection of a non-immersive VR fake mirror can provide information about peripersonal space, which contributes to updating an individual’s body representation from a first-person perspective^21^.

Note that agency and body ownership are two different phenomena^22,23^. Agency requires voluntary action, while body ownership may occur under both voluntary action and passive events^24^. Kalckert and Ehrsson^23^ demonstrated that while asynchrony eliminated both ownership and agency, passive movements abolished the sense of agency but left ownership intact, and, further, incongruent positioning of a virtual hand reduced ownership but did not eliminate agency. In this context, the rubber hand illusion with synchronous visuo-tactile stimulation is a classic example of body-ownership illusion without agency^12^. Contrary to this, a non-immersive VR fake mirror that synchronizes the participant’s voluntary movement with the movement of the virtual body can be an example of the body-ownership illusion with agency^25^.

Previous VR studies in various conditions that manipulate the size of one’s virtual body parts (including arms and feet^26–28^) have shown that, in general, the larger one’s body is perceived, the smaller an object size is considered to be (and vice versa). The rationale for this action-specific perception effect is that the human brain has a tendency to re-calibrate one’s external perception depending on its own body representation (i.e., reference to represent the object in the world in relation to one’s body size). In doing so, the brain combines these perceptions into a symbolically robust idea^29,30^. For instance, if we perceive our referred body size to be large, the visuospatial representation of the external object in the world tends to be shrunken. Therefore, we recognize objects to be smaller than they truly are. In contrast, if we perceive our body size to be small, the visual representation of the external space appears to be enlarged. Therefore, we recognize external objects to be bigger than they actually are^9^.

The body-ownership illusion and its contribution to action-specific perception has been extensively investigated in healthy participants^9,26–28^). However, few studies have studied this topic in neurological patients. Patients with mild cognitive impairment (MCI), who are suspected of having action-specific perception problems due to neurological impairment, are promising study participants^31–33^. Although episodic memory loss has been studied as a major problem in MCI^34–36^, recent studies have focused on the manifestation of impaired multisensory integration^37^. Impaired multisensory integration in MCI patients is associated with general cognitive slowing, inverse effectiveness, an increased temporal window of integration, and deficits in attentional control^16,38–41^. While experiencing the body-ownership illusion, impaired multisensory integration increases the temporal window of integration for body-related cues, which in turn biases our body perception^39,42^. Biased body perception refers to a phenomenon such as perceiving the wrong rubber hand or rubber foot as our own body. For example, using the rubber hand and foot illusion, Hide et al.^43^ showed that older adults with impaired multisensory integration are more susceptible to have biased body perception, which is associated with the construction of a distorted body representation. Note that the body representation is not directly perceived, but is constructed through various cognitive functions, including body perception. A biased body perception in MCI might lead to their characteristic behaviors and actions, such as falls^43,44^. Similarly, Pal et al.^45^ and Quental et al.^46^ reported that dementia patients had perceptual dysfunction in the early stages of dementia, when higher-level cognitive capabilities, such as body transfer illusion (or body-ownership illusion), were first damaged. Overall, this study aims to investigate the action-specific perception impairments of MCI patients by means of body-ownership illusion in a VR environment.

In this study, the following independent participant groups were recruited: 20 healthy young adults, 21 healthy older adults, and 15 elderly patients with MCI. All participants conducted several neuropsychological tests as well as action-specific perception tests in a non-immersive, third-person VR environment (i.e., VR perception tests) in a counter-balanced order. There are several advantages to using a third-person perspective^18,20^. It is low intrusive (i.e., no need to wear a head-mounted display) and enables a full-body representation. Such a representation is difficult to experience in first-person perspective. Furthermore, a third-person perspective is less likely to cause VR sickness, making it more suitable for elderly patients with MCI to use. In the VR perception tests, two virtual body sizes (original body size vs. enlarged body size) were used in order to manipulate the participants’ action capability to interact with circular objects in the VR environment. While using the two different body sizes in VR, the participants were asked to estimate the diameter of a circular object in VR. The diameter of the circle was randomly changed during the experiment. We assessed participants’ original-body action-specific perception and enlarged-body action-specific perception using the original and enlarged sizes of their virtual bodies, respectively. We hypothesized that the MCI patients’ original-body action-specific perception would remain accurate in the VR world. However, we expected more biases from the MCI patients when the enlarged virtual body size was given in the VR world. This biased enlarged-body action-specific perception is expected to provide additional discriminative power for early screening of MCI patients. We suspect that this enlarged-body action-specific perception will offer insight into the mechanisms of complex human perception.

## Results

### Basic demographic characteristics and neuropsychological test results

The participants’ basic demographic characteristics and neuropsychological test results were analyzed using one-way analysis of variance (ANOVA) and post-hoc analyses (see Table 1). Bonferroni-corrected post-hoc analyses showed no statistical differences in age or education level between the MCI patients and the age-matched healthy controls (i.e., healthy older adults). The MCI patients had the poorest performances in most neuropsychological test results compared to those of both healthy young adults and healthy older adults. These tests included the MMSE-DS (effect size *d* = 0.823), DST-F (effect size *d* = 1.007), DST-B (effect size *d* = 0.542), TMT-A (effect size *d* = 0.960), and TMT-B (effect size *d* = 0.870).

**Table 1:** Basic demographic characteristics and neuropsychological test results by group.

	Healthy young adults	Healthy older adults	MCI patients	*p*^†^	*p*^‡^	*p*^§^
**Demographic characteristics**						
Number of participants (male)	20 (10)	21 (13)	15 (5)	–	–	–
Age (years)	27.4 ± 3.0	60.6 ± 6.9	63.3 ± 7.0	< 0.001	< 0.001	0.189
Education level (years)	15.8 ± 0.9	15.0 ± 1.6	14.5 ± 1.6	0.041	0.013	0.279
**Neuropsychological test**						
MMSE-DS	29.1 ± 0.6	28.1 ± 1.3	26.5 ± 2.6	< 0.001	< 0.001	0.004
DST-F (number of correct answers)	13.6 ± 1.7	12.2 ± 2.3	9.8 ± 2.5	< 0.001	< 0.001	0.002
DST-B (number of correct answers)	11.1 ± 3.1	8.7 ± 1.9	7.5 ± 2.6	< 0.001	< 0.001	0.193
TMT-A (time to completion, seconds)	20.3 ± 5.3	31.7 ± 7.6	40.3 ± 10.6	< 0.001	< 0.001	0.002
TMT-A (number of errors)	0.2 ± 0.5	0.2 ± 0.4	0.3 ± 0.5	0.729	0.463	0.855
TMT-B (time to completion, seconds)	41.2 ± 10.1	60.9 ± 12.7	73.7 ± 17.2	< 0.001	< 0.001	0.006
TMT-B (number of errors)	0.3 ± 0.6	1.0 ± 0.9	0.7 ± 1.0	0.028	0.112	0.372

Values are expressed as means ± SDs.

*MMSE-DS* mini mental state examination-dementia screening, *DST-F* digit span test-forward, *DST-B* digit span test-backward, *TMT-A* trail making test-A, *TMT-B* trail making test-B.

^†^ANOVA analysis, healthy young adults v. healthy older adults v. MCI patients.

^‡^Post-hoc analysis, healthy young adults v. MCI patients.

^§^Post-hoc analysis, healthy older adults v. MCI patients.

### VR perception test results

The paired sample t-tests showed that all three groups perceived the diameter of the circle (the diameter of a circle was randomly presented) to be significantly smaller in the enlarged virtual body condition than they did in the original virtual body condition. In the enlarged virtual body condition, both healthy young adults (− 0.28 ± 0.51 cm; t(19) = 2.442, *p* = 0.025, effect size *d* = 0.576) and healthy older adults (− 0.40 ± 0.42 cm; t(20) = 4.364, *p* < 0.001, effect size *d* = 0.962) perceived the diameter of the circle to be smaller than that of the original virtual body condition. However, compared to both healthy young adults and healthy older adults, the MCI patients perceived objects to be much smaller in the enlarged virtual body condition than they were in the original virtual body condition (− 0.86 ± 0.50 cm; t(14) = 6.685, *p* < 0.001, effect size *d* = 2.087).

**Figure 1:**
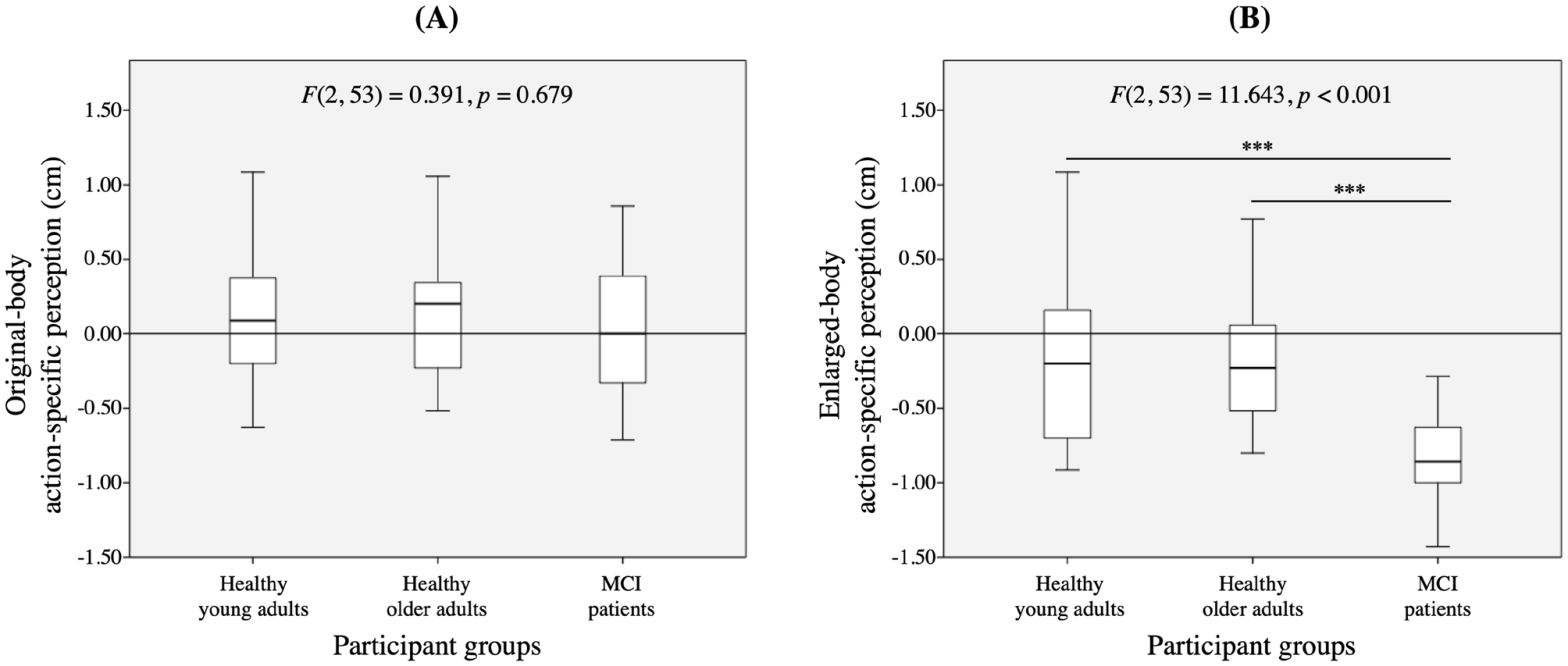
Box plots for virtual reality perception test results by group. (**A**) Original-body action-specific perception. (**B**) Enlarged-body action-specific perception.

The VR perception test results were analyzed by one-way ANOVA (see Table 2, Fig. 1). There was no statistically significant difference in the original-body action-specific perception among the three groups, F(2, 53) = 0.391, *p* = 0.679. However, the MCI patients had a higher level of biased enlarged-body action-specific perception (− 0.9 ± 0.3 cm) than did healthy young adults (− 0.2 ± 0.5 cm) and healthy older adults (− 0.3 ± 0.4 cm), F(2, 53) = 11.643, *p* < 0.001, effect size *d* = 1.657. Bonferroni-corrected post-hoc analyses confirmed this assertion.

**Table 2:** The virtual reality perception test results by group.

	Healthy young adults	Healthy older adults	MCI patients	*p*^†^	*p*^‡^	*p*^§^
**Virtual reality perception test**						
Original-body action-specific perception (cm)	0.1 ± 0.5	0.1 ± 0.4	0.01 ± 0.5	0.679	0.480	0.405
Enlarged-body action-specific perception (cm)	− 0.2 ± 0.5	− 0.3 ± 0.4	− 0.9 ± 0.3	< 0.001	< 0.001	< 0.001

Values are expressed as means ± SDs.

^†^ANOVA analysis, healthy young adults v. healthy older adults v. MCI patients.

^‡^Post-hoc analysis, healthy young adults v. MCI patients.

^§^Post-hoc analysis, healthy older adults v. MCI patients.

Immediately after each virtual body size condition, participants verbally rated a question—*I felt that the virtual body I saw when looking at myself on the screen was my own body* (modified from an original question by Ref.^12^—to report their sense of body ownership in VR on a 1–7 Likert scale (1 means “strongly disagree” and 7 means “strongly agree”). As shown in Table 3, all participants reported high levels of the sense of body ownership in the VR perception test. Bonferroni-corrected analyses showed no statistical differences in the sense of body ownership between participant groups.

**Table 3:** The sense of body ownership by group in each virtual reality perception test condition.

	Healthy young adults	Healthy older adults	MCI patients	*p*^†^
**Virtual reality perception test**				
Original virtual body condition	6.6 ± 0.5	6.5 ± 0.5	6.5 ± 0.5	0.891
Enlarged virtual body condition	6.6 ± 0.5	6.6 ± 0.5	6.6 ± 0.5	0.959

Values are expressed as means ± SDs.

^†^ANOVA analysis, healthy young adults v. healthy older adults v. MCI patients.

### Correlation between VR perception test and neuropsychological tests

A Pearson correlation analysis was performed to examine the relationship between the VR perception test results and the neuropsychological test results. A Bonferroni-adjusted alpha level was applied for multiple correlation tests. The original-body action-specific perception (i.e., the original virtual body condition) demonstrated no correlation with the neuropsychological test results (*p* > 0.05). The enlarged-body action-specific perception (i.e., the enlarged virtual body condition) was weakly correlated with the overall cognitive function, such as the MMSE-DS (*r* = 0.275, *p* = 0.040, effect size *d* = 0.572), DST-F (*r* = 0.273, *p* = 0.042, effect size *d* = 0.568), TMT-A (*r* =  − 0.274, *p* = 0.041, effect size *d* = 0.570), and TMT-B (*r* =  − 0.270, *p* = 0.044, effect size *d* = 0.561). These findings suggest that the enlarged-body action-specific perception test can provide additional independent information.

### Discriminative power of the VR perception test for screening MCI patients

**Figure 2:**
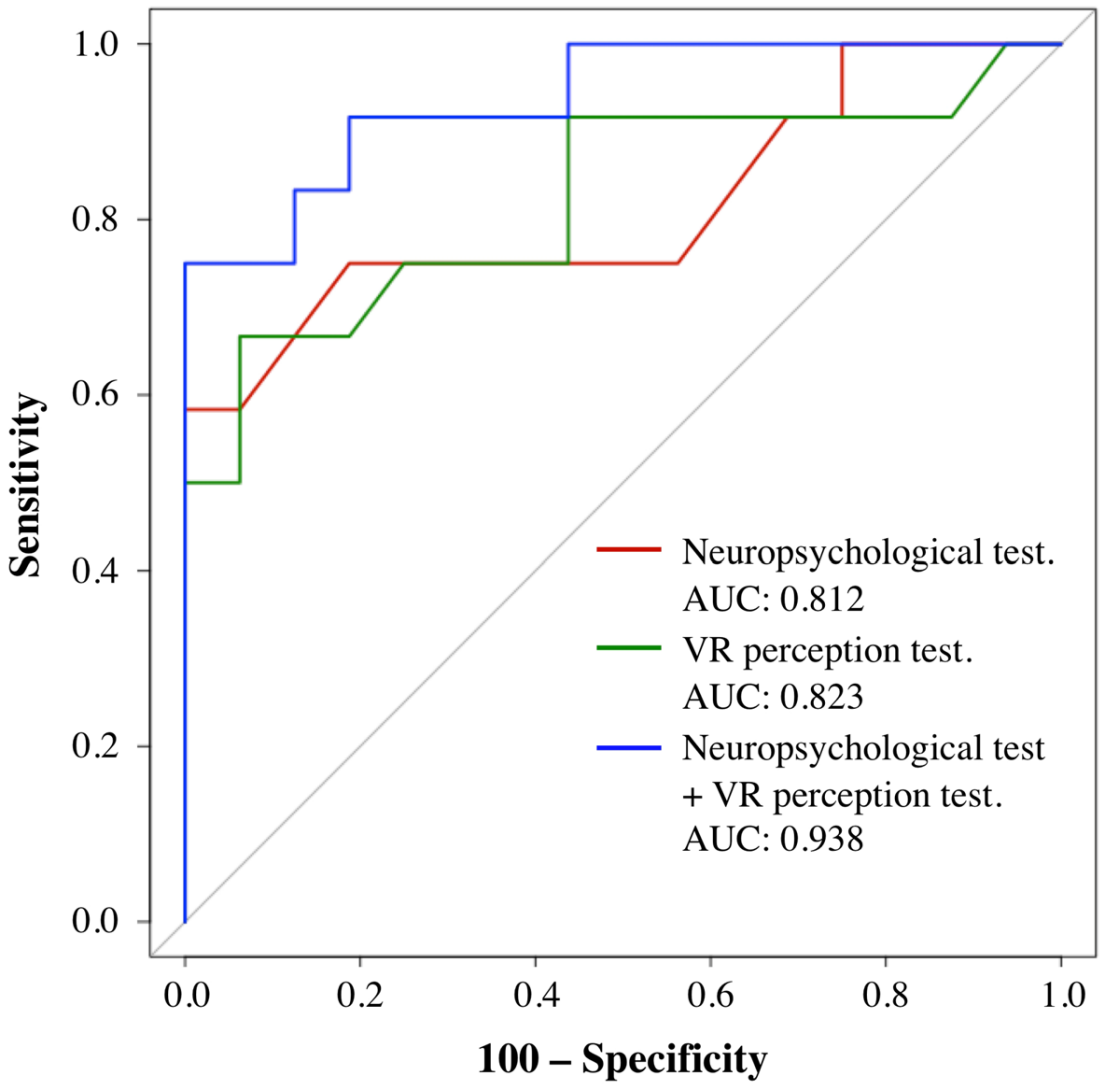
Comparison of receiver operating characteristic (ROC) curves. The area under the ROC curve (AUC) is shown. The best diagnostic performance was obtained by performing the neuropsychological test and the VR perception test concurrently. The neuropsychological test (TMT-A, with a cut-off value of 34.5) and the VR perception test (enlarged-body action-specific perception, with a cut-off value of − 0.7 cm) distinguished between the patients with MCI and the other groups with 75.0% sensitivity and 100.0% specificity.

A forward stepwise linear discriminant analysis (LDA) with a leave-one-out cross-validation procedure was applied to examine the discriminative power of our VR perception test and the conventional neuropsychological tests. As shown in Table 4 and Fig. 2, the MCI patients were best discriminated from the healthy older adults when both a neuropsychological test (*inter alia,* TMT-A) and our VR perception test (*inter alia*, enlarged-body action-specific perception) were performed (89.3% accuracy, 75.0% sensitivity, 100.0% specificity, and 87.5% balanced accuracy). However, the neuropsychological tests and our VR perception tests on their own were only able to classify a fraction of the MCI patients. Therefore, the inclusion of our VR perception test provides additional discriminative power for the early screening of MCI patients.

**Table 4:** Overall classification performance of the discriminant analysis model to diagnose MCI.

Steps	Predictor variables	Wilks’ Lambda (*p*)	Accuracy (%)	Sensitivity (%)	Specificity (%)	Balanced accuracy (%)
Step 1	Neuropsychological test (TMT-A)	0.808 (0.008)	78.5	75.0	81.3	78.1
Step 2	Virtual reality perception test (enlarged-body action-specific perception)	0.702 (< 0.001)	82.1	66.7	93.8	80.3
Step 3	Neuropsychological test (TMT-A) + virtual reality perception test (enlarged-body action-specific perception)	0.510 (< 0.001)	89.3	75.0	100.0	87.5

## Discussion

The main goal of this study was to examine the effect of action-specific perception in MCI patients. Our proposed VR perception test was used to quantitatively evaluate the action-specific perception of MCI patients under both the original virtual body size condition and the enlarged virtual body size condition. In the original virtual body size condition, there were no statistical differences in original-body action-specific perception between MCI patients and healthy controls. These results suggest that the action-specific perception of MCI patients remains accurate in their original body size. However, the action-specific perception in MCI patients seemed to be impaired when the virtual body size was enlarged. This finding suggests that the assessment of enlarged-body action-specific perception in VR can be used to identify MCI patients.

These results are comparable to recent neuroimaging studies, which showed that neural pathways play an important role in the association between actions and perception^31–33^. MCI patients perceived the same object to be much smaller than that of healthy controls when their virtual body size was enlarged. These results can be interpreted to mean that the visual pathways for action are impaired in MCI patients (i.e., impaired action-specific perception^2^. In a similar vein, Murray et al.^37^ demonstrated impaired multisensory integration function in patients with MCI. Impaired multisensory integration biases perception of body representation^43^. In other words, in the enlarged virtual body size condition, impaired multisensory integration in MCI patients could be associated with a misrepresentation of their body, which also appears to be associated with perception of the same object as much smaller than that of healthy controls.

Action-specific perception impairments in MCI patients can be further understood through the interaction between two different types of body representation, the body image and the body schema (for a review, see^47,48^. The distinction between the body image and the body schema owes much to the perception–action model of vision^2,3^). The body image is responsible for visuo-spatial representation of the body (e.g., body part judgments) and the body schema is responsible for action-oriented sensorimotor representation of the body (e.g., posture, limb size, and strength). Pitron et al.^47^ proposed a feedback loop in which the body image acts as a prior for the body schema. The body image is compared to the body schema, and if there is too much discrepancy between the two (given a certain threshold) and this discrepancy remains over time, then the body image is used to (re)calibrate the body schema. When this (re)calibration is damaged, body representation is distorted, such as in Alice in Wonderland Syndrome (i.e., distorted awareness of the size, mass, shape of the body, or its position in space) and macro/microsomatognosia (i.e., distorted awareness of the size of the whole body or of body parts, for a review, see Ref.^48^. Our findings can be explained through a malfunction in the feedback loop between the body image and the body schema. For example, the reason MCI patients perceive the same object to be much smaller than that of healthy controls in the enlarged virtual body size condition may be due to the distortion that occurs when (re)calibrating the body schema based on the body image. The MCI patients’ illusion of having a large body appears to (re)calibrate the body schema more easily than that of healthy controls, resulting in a distorted awareness of the size of external objects (i.e., much smaller than they really are).

Although the existing neuropsychological tests can diagnose some MCI patients, these tests have significantly improved predictive power (i.e., accuracy, sensitivity, specificity, and balanced accuracy) when they were used in conjunction with our VR perception test (i.e., enlarged-body action-specific perception). Interestingly, the combination of TMT-A and the VR perception test showed higher discriminatory power in early screening of MCI patients than using MMSE-DS. This result suggests that assessment of psychomotor speed and action-specific perception together characterizes MCI patients better than assessing general cognitive function. MCI diagnosis through action-specific perception assessment in VR has not been empirically tested before. Therefore, our findings can be utilized to optimize the diagnosis of MCI by combining neuropsychological tests with the VR perception test (e.g.^49,50^). Furthermore, a VR perception test can be used for MCI treatment. For example, the full-body illusion in VR can be utilized to improve executive functioning and increase prefrontal cortex activity in the elderly^51^.

The assessment of action-specific perception should be distinguished from the Ebbinghaus illusion, which is an optical illusion of relative size perception^52^. Action-specific perception represents a visual pathway for action (i.e., vision-for-action), whereas, the Ebbinghaus illusion relates to a visual pathway for perception (i.e., vision-for-perception). Goodale and Milner^2^ demonstrated that visually guided movements are largely immune to the perceptually compelling changes in size produced by pictorial illusions. Haffenden et al.^53^ further showed that the size-contrast illusion elicited by the Ebbinghaus display does not affect grasp scaling. That is, our VR perception test can be free from the Ebbinghaus illusion as participants were instructed to grab a virtual circle with their virtual body. Subsequent studies evaluating the Ebbinghaus illusion in patients with MCI will further understand the impact of neurological impairment on separate visual pathways for perception and action.

The added value of our study comes from assessing MCI patients based on their ability to relate perception and action capabilities within a VR environment (i.e., the affordances of an enlarged virtual body size). A person’s action-specific perception is generally set by one’s own ability to understand the environment around them and its properties as well as their own properties (i.e., body representation). Most of our daily activities reflect a perfect adjustment between the perceptions of potential actions and our actual actions. However, when this action-specific perception is impaired, there is a significant perceptual and cognitive gap between what they believe they can do and what they can actually do. For instance, falls in older adults have been mainly believed to arise from physical capabilities in action (e.g., weaker muscles, reduced postural abilities, and unstable equilibration). However, many empirical studies have suggested that one’s cognitive capability to scale down or up his/her perceptual estimation of the external world is an early marker of fall risk (e.g.^43,54–56^. The VR perception tests introduced in this study are used in this regard in the early diagnosis or screening for MCI. Indeed, this VR technology can quickly provide a variety of sensory stimuli (for a meta-review, see Ref.^57^, and therefore can have significant implications in an evidence-based medical assessment^58^. Our study also proposes that VR technology explores a new dimension of patient data (i.e., action-specific perception). With this new data, we can classify patients using many new machine learning techniques (e.g.^16,38,59,60^), in contrast to traditional assessments that use interviews and paper-and-pencil tests.

This study has several limitations. First, although the VR perception test was able to discriminate the MCI patients from healthy controls, further clinical studies are needed to validate our findings. A second limitation is that we are unable to explain the MCI-related pathophysiological mechanisms by which patients have impaired action-specific perception. Therefore, future studies are needed to evaluate the clinical validity and the relationship between cognitive dysfunction and impaired action-specific perception. For instance, future studies must address various action-specific perceptions (e.g., relocating objects apart from simple grabbing) from the perspective of a different enlarged virtual body (e.g., besides a 20% up- or down-scale in size of the virtual body). Additionally, further experimental studies of how MCI patients’ body representation changes in VR will provide a deeper understanding of impaired action-specific perception. Impaired body representation can lead to various disorders of bodily awareness^48^. Therefore, by studying how MCI patients’ body representation is changed in VR, one may be able to understand how the brain represents the body and how it represents it in a different way depending on its action capabilities (i.e., action-specific perception).

Overall, this study is the first to examine the action-specific perception impairments of MCI patients. We demonstrated that the illusion of having a large virtual body biases action-specific perception in MCI patients and consequently causes MCI patients to perceive surrounding objects much smaller than their actual size. Therefore, we believe that the VR perception tests introduced in this study will provide important discriminative features about MCI patients beyond those of conventional neuropsychological tests, which are highly correlated with general intelligence (e.g. Huygelier et al.^61^).

## Methods

### Participants

We recruited 20 healthy young adults (10 males and 10 females; average age is 27.4 ± 3.0 years; range is 20–32 years), 21 healthy older adults (13 males and 8 females; average age is 60.6 ± 6.9 years; range is 50–70 years), and 15 elderly patients with MCI (5 males and 10 females; average age is 63.3 ± 7.0 years; range is 51–69 years). All participants had normal or corrected eyesight. Written informed consent form was obtained from each participant after the experimental procedure was explained to them. For MCI patients, additional informed consent was required from their guardian for patient safety. All experimental protocols were approved by the Hanyang University Institutional Review Board (HYI-18-142). All methods were carried out in accordance with the Declaration of Helsinki.

Healthy young adults were sent from a community health promotion center. Healthy older adults were recruited from a pool of community volunteers with no reported health problems. MCI patients were randomly selected from outpatients at the department of neurology in a general hospital. All participants volunteered to participate in this study. Two independent neurologists (with 17 and 20 years of experience) completed the neuropsychological tests, physical examinations, and medical histories. These two neurologists diagnosed MCI using the criteria described by Albert et al.^34^. Participants who abused drugs or consumed alcohol heavily within four weeks of the start date of the study were excluded (based on the clinical interview). Other exclusion criteria included a history of brain surgery and a history of neurological/psychiatric diseases, such as Parkinson’s disease, Huntington’s disease, epilepsy, multiple sclerosis, depression, and anxiety disorders.

### Procedure

All the experiments (i.e., neuropsychological tests and the VR perception test) were supervised by a specially trained psychologist on a one-to-one basis. The with-in order of the neuropsychological tests (MMSE-DS, DST-F, DST-B, TMT-A, and TMT-B; see below for details) and the VR perception test environments (i.e., original body size and enlarged body size) were counter-balanced. The latter (VR perception tests) followed the former (neuropsychological tests). Before the VR perception tests, all of the participants had a 10-min training session to get accustomed to interacting with their virtual body and the virtual objects in the 2D VR environment. During the VR perception tests, participants verbally rated a question about their sense of body ownership in VR (I felt that the virtual body I saw when looking at myself on the screen was my own body) on a 1–7 Likert scale (1 means “strongly disagree” and 7 means “strongly agree”). No participant complained about VR sickness.

### Neuropsychological tests

A total of five neuropsychological tests were administered to the participants. These tests included: (i) mini mental state examination-dementia screening (MMSE-DS) to assess general cognitive function^62^, (ii) digit span test-forward (DST-F), (iii) digit span test-backward (DST-B) for executive function^63^, (iv) trail making test-A (TMT-A), and (v) trail making test-B (TMT-B) for psychomotor speed^63^.

### VR perception test

**Figure 3:**
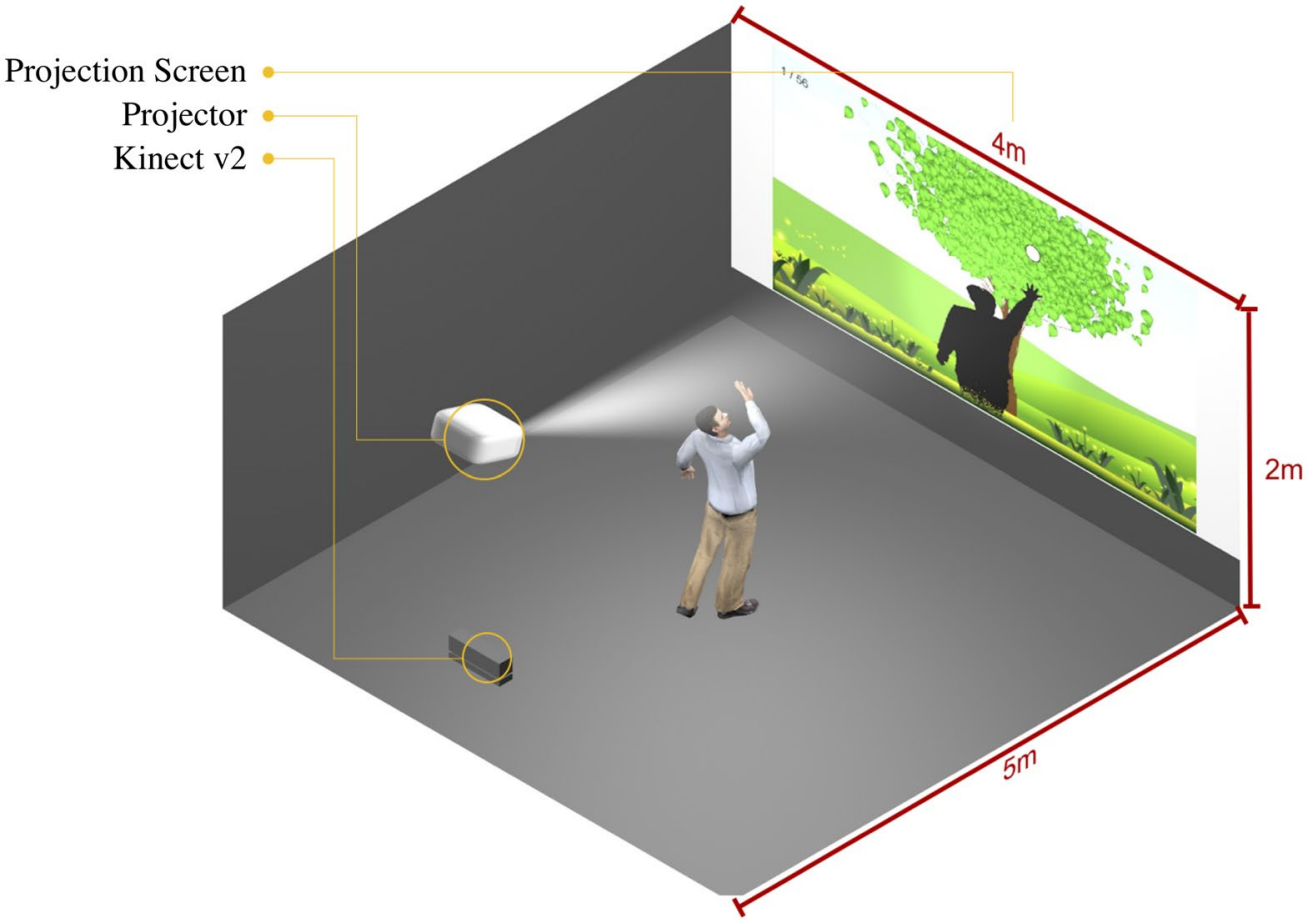
Virtual reality perception test setting. The participant’s full body movements were tracked by an optical tracking system (Kinect v2) and mapped in real-time to the movements of the virtual body on a large screen.

We set up the VR perception tests to manipulate the size of the participants’ virtual body while they interacted with virtual objects. As shown in Fig. 3, the VR perception test was conducted in a full tracking space with a large projection screen (4 × 2 × 5 m^3^). The participant’s full body movements were tracked using an optical tracking system (Kinect v2, Microsoft, USA). These movements were mapped onto the virtual body on the projected screen (i.e., a non-immersive VR fake mirror^18,20^). In order to induce the body-ownership illusion, the movements of the virtual body were synchronous with those of each participant in real time (i.e., visuo-motor synchrony^23,64^.

**Figure 4:**
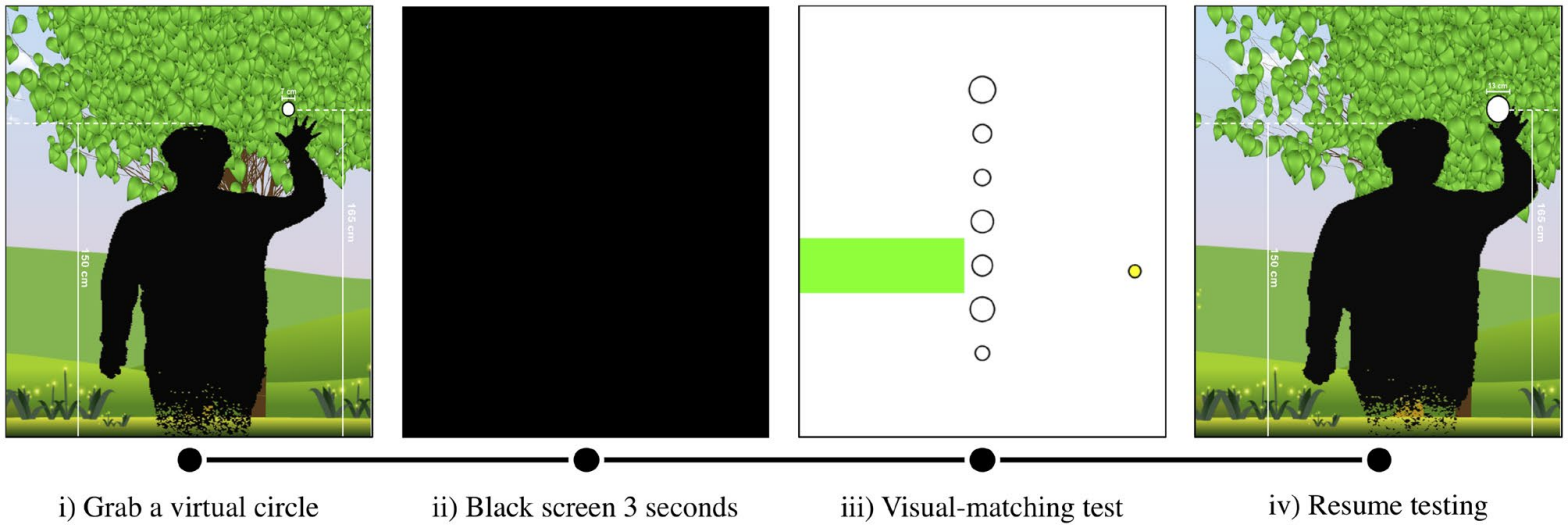
Procedures for the VR perception tests. (**i**) Participants grab a virtual circle. (**ii**) A black screen appears for three seconds. (**iii**) Participants select a circle (i.e., grab a circle) that visually matches the circle they grabbed. (**iv**) Participants continue the test.

Figure 4 shows the sequence in which the VR perception tests are performed. First, (i) the participants are instructed to grab a virtual circle with their virtual body (note that all participants successfully grabbed the circle in each attempt). One circle is randomly selected and displayed out of seven possibilities (with the diameters of 7 cm, 8 cm, 9 cm, 10 cm, 11 cm, 12 cm or 13 cm). The circle is displayed above the participant’s head, so they can easily recognize the objects to grasp. Next, (ii) after five seconds, a black screen appeared for three seconds to remove the effects of perceptual memory. Following this, (iii) the black screen disappears, and the participant is given a multiple-choice question consisting of seven circles with different diameters. The participants are instructed to choose a circle (i.e., grab a circle) that visually matches the circle they just grabbed. This is a visual-matching test. In the fourth step, (iv) the participants continue the VR perception test until they are notified to stop.

**Figure 5:**
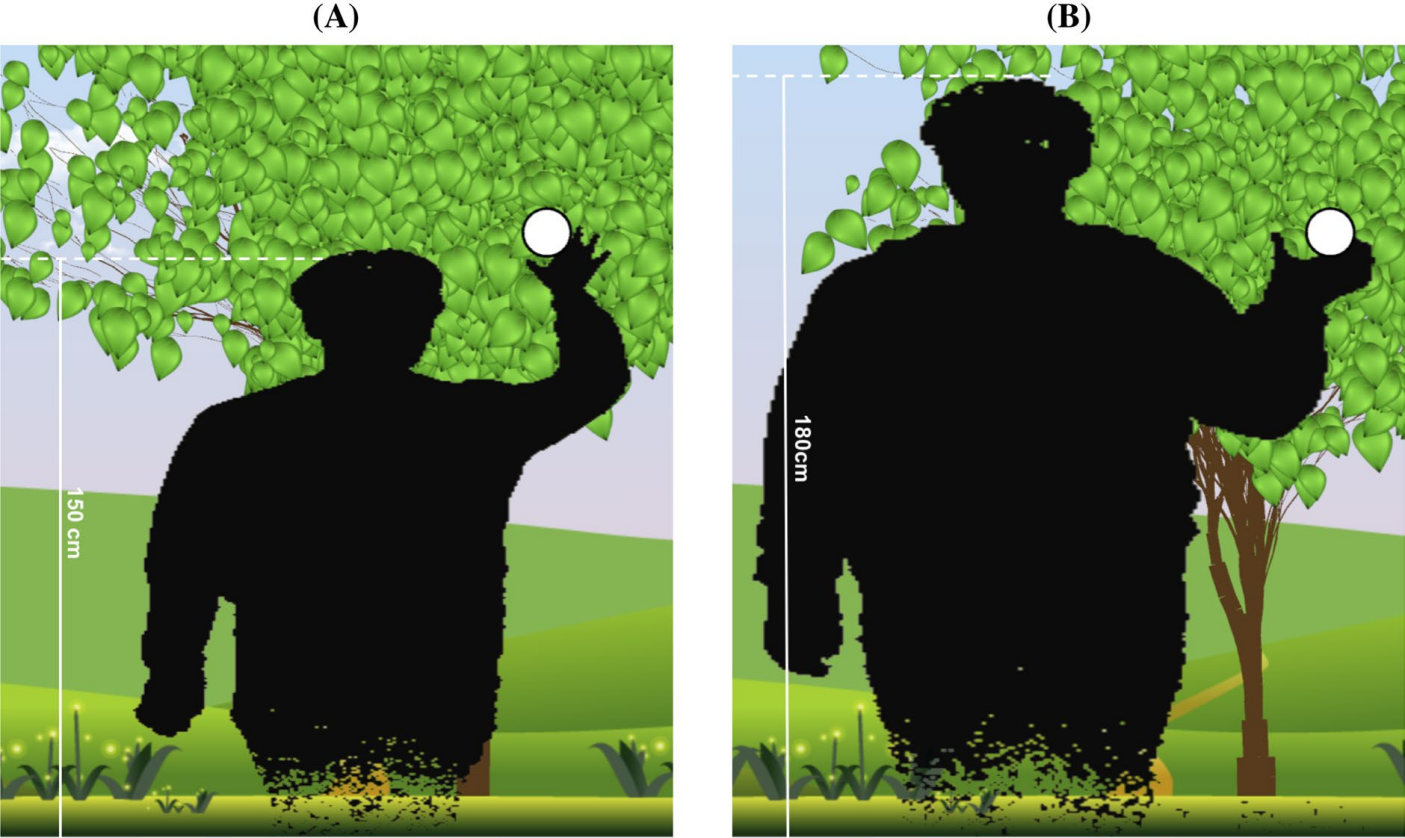
Two different body size conditions in the virtual reality perception tests. (**A**) An original virtual body size condition. (**B**) An enlarged virtual body size condition.

The VR perception tests were administered using the following two different virtual body size conditions: the original virtual body size and the enlarged virtual body size (see Fig. 5). In the original virtual body size condition, all of the participants saw the same-sized virtual body with their real body. In the enlarged virtual body size condition, the virtual body size was enlarged by 20%. The sizes of other objects (e.g., circles to grasp, trees, leaves, and etc.) in the VR world were not enlarged.

The participant’s responses were measured by the chosen size of the circle in the visual-matching test (e.g., if a participant selected 8 cm when the correct answer was 9 cm, the response was calculated as 8 − 9 =  − 1 cm). A total of seven trials were randomly made using the original virtual body size condition. The average of these seven trials was the “original-body action-specific perception” measure. Note that “original-body action-specific perception” represents the participant’s action-specific perception in the original virtual body size condition. The same procedures were repeated using the enlarged virtual body size condition (i.e., a total of seven additional trials were carried out). The average of these seven trials was the “enlarged-body action-specific perception” measure. Both conditions were conducted in a random order to avoid order effects.

### Statistical analysis

To test both whether action-specific perception was impaired or not in the pre-dementia MCI patients and the validity of the VR perception tests as a screening tool, several statistical methods were applied using IBM SPSS Statistics 21. Prior to the main statistical analyses, a one-way analysis of variance (ANOVA) was used to examine the group differences in basic demographic characteristics and neuropsychological test results. Second, paired sampled t-tests were used to compare the differences between the “original-body action-specific perception” in the original virtual body size condition and the “enlarged-body action-specific perception” in the enlarged virtual body size condition. ANOVA was then used to examine both the group differences in the VR perception test results and the sense of body ownership in VR. For all ANOVAs, a Bonferroni-correction was used for post-hoc analyses and effect sizes were reported by Cohen’s *d*. Third, a Pearson correlation analysis for a validity check was conducted between the neuropsychological tests and the VR perception test. Finally, a forward stepwise Linear Discriminant Analysis (LDA) with cross-validation was performed to identify the discriminative power of the VR perception test (and if this can be seen as a potential diagnostic tool for patients with MCI).

## Data Availability

All study-associated data and material is stored at the Department of Applied Artificial Intelligence, Seoul National University of Science and Technology, and access is available by request.

## References

[1] Noë A (2002). Is the visual world a grand illusion?. J. Conscious. Stud..

[2] Goodale MA, Milner AD (1992). Separate visual pathways for perception and action. Trends Neurosci..

[3] Goodale MA (2011). Transforming vision into action. Vis. Res..

[4] Gibson JJ (2014). The Ecological Approach to Visual Perception.

[5] Witt JK (2011). Action’s effect on perception. Curr. Dir. Psychol. Sci..

[6] Witt JK, Riley MA (2014). Discovering your inner Gibson: Reconciling action-specific and ecological approaches to perception–action. Psychon. Bull. Rev..

[7] Linkenauger SA, Witt JK, Proffitt DR (2011). Taking a hands-on approach: Apparent grasping ability scales the perception of object size. J. Exp. Psychol. Hum. Percept. Perform..

[8] Witt JK, Linkenauger SA, Wickens C (2016). Action-specific effects in perception and their potential applications. J. Appl. Res. Mem. Cogn..

[9] Van Der Hoort B, Ehrsson HH (2016). Illusions of having small or large invisible bodies influence visual perception of object size. Sci. Rep..

[10] Slater M, Pérez Marcos D, Ehrsson H, Sanchez-Vives MV (2009). Inducing illusory ownership of a virtual body. Front. Neurosci..

[11] Slater M, Sanchez-Vives MV (2014). Transcending the self in immersive virtual reality. Computer.

[12] Botvinick M, Cohen J (1998). Rubber hands ‘feel’ touch that eyes see. Nature.

[13] Kammers MP, de Vignemont F, Verhagen L, Dijkerman HC (2009). The rubber hand illusion in action. Neuropsychologia.

[14] Kammers MP, Kootker JA, Hogendoorn H, Dijkerman HC (2010). How many motoric body representations can we grasp?. Exp. Brain Res..

[15] Pan X, Hamilton AFDC (2018). Why and how to use virtual reality to study human social interaction: The challenges of exploring a new research landscape. Br. J. Psychol..

[16] Seo K, Kim JK, Oh DH, Ryu H, Choi H (2017). Virtual daily living test to screen for mild cognitive impairment using kinematic movement analysis. PLoS ONE.

[17] Slater M, Spanlang B, Sanchez-Vives MV, Blanke O (2010). First person experience of body transfer in virtual reality. PLoS ONE.

[18] Lugrin, J. L., Zilch, D., Roth, D., Bente, G. & Latoschik, M. E. Facebo: Real-time face and body tracking for faithful avatar synthesis. In *2016 IEEE Virtual Reality (VR)*, 225–226 (IEEE, 2016).

[19] Seo K, Kim J, Ryu H, Jang S, Holzinger A, Ziefle M, Röcker C (2014). ‘RehabMaster TM’: A pervasive rehabilitation platform for stroke patients and their caregivers. Pervasive Health. Human–Computer Interaction Series.

[20] Lugrin, J. L., Obremski, D., Roth, D. & Latoschik, M. E. Audio feedback and illusion of virtual body ownership in mixed reality. In *Proc. 22nd ACM Conference on Virtual Reality Software and Technology*, 309–310 (2016).

[21] Preston C, Kuper-Smith BJ, Ehrsson HH (2015). Owning the body in the mirror: The effect of visual perspective and mirror view on the full-body illusion. Sci. Rep..

[22] Kalckert A, Ehrsson HH (2012). Moving a rubber hand that feels like your own: A dissociation of ownership and agency. Front. Hum. Neurosci..

[23] Kalckert A, Ehrsson HH (2014). The moving rubber hand illusion revisited: Comparing movements and visuotactile stimulation to induce illusory ownership. Conscious. Cogn..

[24] Tsakiris M, Schütz-Bosbach S, Gallagher S (2007). On agency and body-ownership: Phenomenological and neurocognitive reflections. Conscious. Cogn..

[25] Dewez, D., Hoyet, L., Lécuyer, A. & Argelaguet Sanz, F. A. Towards “avatar-friendly” 3D manipulation techniques: Bridging the gap between sense of embodiment and interaction in virtual reality. In *Proc. 2021 CHI Conference on Human Factors in Computing Systems*, 1–14 (2021).

[26] Jun E, Stefanucci JK, Creem-Regehr SH, Geuss MN, Thompson WB (2015). Big foot: Using the size of a virtual foot to scale gap width. ACM Trans. Appl. Percept..

[27] Linkenauger SA, Bülthoff HH, Mohler BJ (2015). Virtual arm’s reach influences perceived distances but only after experience reaching. Neuropsychologia.

[28] van der Hoort B, Guterstam A, Ehrsson HH (2011). Being Barbie: The size of one’s own body determines the perceived size of the world. PLoS ONE.

[29] Ernst MO, Banks MS (2002). Humans integrate visual and haptic information in a statistically optimal fashion. Nature.

[30] Ernst MO, Bülthoff HH (2004). Merging the senses into a robust percept. Trends Cogn. Sci..

[31] Burunat I, Tsatsishvili V, Brattico E, Toiviainen P (2017). Coupling of action-perception brain networks during musical pulse processing: Evidence from region-of-interest-based independent component analysis. Front. Hum. Neurosci..

[32] Christensen A, Giese MA, Sultan F, Mueller OM, Goericke SL, Ilg W, Timmann D (2014). An intact action-perception coupling depends on the integrity of the cerebellum. J. Neurosci..

[33] Hutchison RM, Gallivan JP (2018). Functional coupling between frontoparietal and occipitotemporal pathways during action and perception. Cortex.

[34] Albert MS, DeKosky ST, Dickson D, Dubois B, Feldman HH, Fox NC (2011). The diagnosis of mild cognitive impairment due to Alzheimer’s disease: Recommendations from the National Institute on Aging-Alzheimer’s Association workgroups on diagnostic guidelines for Alzheimer's disease. Alzheimers Dement..

[35] American Psychiatric Association (2013). Diagnostic and statistical manual of mental disorders. BMC Med..

[36] Petersen RC, Doody R, Kurz A, Mohs RC, Morris JC, Rabins PV (2001). Current concepts in mild cognitive impairment. Arch. Neurol..

[37] Murray MM, Eardley AF, Edginton T, Oyekan R, Smyth E, Matusz PJ (2018). Sensory dominance and multisensory integration as screening tools in aging. Sci. Rep..

[38] Seo K, Jun DW, Kim JK, Ryu H (2017). Multi-sensory integration impairment in patients with minimal hepatic encephalopathy. Sci. Rep..

[39] Chan S, Kaiser J, Brandl M, Matura S, Prvulovic D, Hogan M, Naumer M (2015). Expanded temporal binding windows in people with mild cognitive impairment. Curr. Alzheimer Res..

[40] Mozolic JL, Hugenschmidt CE, Peiffer AM, Laurienti PJ, Murray MM, Wallace MT (2012). Multisensory integration and aging. The Neural Bases of Multisensory Processes.

[41] Wu J, Yang J, Yu Y, Li Q, Nakamura N, Shen Y (2012). Delayed audiovisual integration of patients with mild cognitive impairment and Alzheimer's disease compared with normal aged controls. J. Alzheimers Dis..

[42] Maselli A, Kilteni K, López-Moliner J, Slater M (2016). The sense of body ownership relaxes temporal constraints for multisensory integration. Sci. Rep..

[43] Hide M, Ito Y, Kuroda N, Kanda M, Teramoto W (2021). Multisensory integration involved in the body perception of community-dwelling older adults. Sci. Rep..

[44] Tezuka K, Meguro K, Akanuma K, Tanaka N, Ishii H, Yamaguchi S (2013). Overestimation of self-reported activities of daily living in vascular dementia patients with a right hemisphere lesion. J. Stroke Cerebrovasc. Dis..

[45] Pal A, Biswas A, Pandit A, Roy A, Guin D, Gangopadhyay G, Senapati AK (2016). Study of visuospatial skill in patients with dementia. Ann. Indian Acad. Neurol..

[46] Quental NBM, Brucki SMD, Bueno OFA (2013). Visuospatial function in early Alzheimer’s disease—The use of the visual object and space perception (VOSP) battery. PLoS ONE.

[47] Pitron V, Alsmith A, de Vignemont F (2018). How do the body schema and the body image interact?. Conscious. Cogn..

[48] De Vignemont F (2010). Body schema and body image—Pros and cons. Neuropsychologia.

[49] Panegyres PK, Berry R, Burchell J (2016). Early dementia screening. Diagnostics.

[50] Robinson L, Tang E, Taylor JP (2015). Dementia: Timely diagnosis and early intervention. BMJ.

[51] Burin D, Kawashima R (2021). Repeated exposure to illusory sense of body ownership and agency over a moving virtual body improves executive functioning and increases prefrontal cortex activity in the elderly. Front. Hum. Neurosci..

[52] Roberts B, Harris MG, Yates TA (2005). The roles of inducer size and distance in the Ebbinghaus illusion (Titchener circles). Perception.

[53] Haffenden AM, Schiff KC, Goodale MA (2001). The dissociation between perception and action in the Ebbinghaus illusion: Nonillusory effects of pictorial cues on grasp. Curr. Biol..

[54] Caffier D, Luyat M, Crémoux S, Gillet C, Ido G, Barbier F, Naveteur J (2019). Do older people accurately estimate the length of their first step during gait initiation?. Exp. Aging Res..

[55] Luyat M, Domino D, Noël M (2008). Can overestimating one's own capacities of action lead to fall? A study on the perception of affordance in the elderly. Psychol. Neuropsychiatr. Vieil..

[56] Stafford J, Whyatt C, Craig CM (2019). Age-related differences in the perception of gap affordances: Impact of standardized action capabilities on road-crossing judgements. Accid. Anal. Prev..

[57] Neguț A, Matu SA, Sava FA, David D (2016). Virtual reality measures in neuropsychological assessment: A meta-analytic review. Clin. Neuropsychol..

[58] Ma M, Jain LC, Anderson P (2014). Virtual, Augmented Reality and Serious Games for Healthcare.

[59] Riener R, Harders M, Riener R, Harders M (2012). Virtual reality for rehabilitation. Virtual Reality in Medicine.

[60] Seo K, Lee A, Kim J, Ryu H, Choi H (2018). Measuring the kinematics of daily living movements with motion capture systems in virtual reality. JoVE.

[61] Huygelier H, Van der Hallen R, Wagemans J, De-Wit L, Chamberlain R (2018). The Leuven embedded figures test (L-EFT): Measuring perception, intelligence or executive function?. PeerJ.

[62] Han JW, Kim TH, Jhoo JH, Park JH, Kim JL, Ryu SH (2010). A normative study of the mini-mental state examination for dementia screening (MMSE-DS) and its short form (SMMSE-DS) in the Korean elderly. J. Korean Geriatr. Psychiatry.

[63] Nasreddine ZS, Phillips NA, Bédirian V, Charbonneau S, Whitehead V, Collin I (2005). The Montreal cognitive assessment, MoCA: A brief screening tool for mild cognitive impairment. J. Am. Geriatr. Soc..

[64] Dummer T, Picot-Annand A, Neal T, Moore C (2009). Movement and the rubber hand illusion. Perception.

